# Clinical research in Finland in 2002 and 2007: quantity and type

**DOI:** 10.1186/1478-4505-11-17

**Published:** 2013-05-16

**Authors:** Elina Hemminki, Jorma Virtanen, Piret Veerus, Elena Regushevskaya

**Affiliations:** 1 National Institute for Health and Welfare, P.O. Box 30, Helsinki 00271, Finland; 2 Hjelt Institute/Department of Public Health, University of Helsinki, P.O. Box 41, Helsinki 00014, Finland; 3 National Institute for Health Development, Hiiu 42, Tallinn 11619, Estonia; 4 University of Oulu, Faculty of Medicine, P.O. Box 5281, Oulu 90014, Finland

**Keywords:** Clinical research, Drug trials, Finland, Sponsoring

## Abstract

**Background:**

Regardless of worries over clinical research and various initiatives to overcome problems, few quantitative data on the numbers and type of clinical research exist. This article aims to describe the volume and type of clinical research in 2002 and 2007 in Finland.

**Methods:**

The research law in Finland requires all medical research to be submitted to regional ethics committees (RECs). Data from all new projects in 2002 and 2007 were collected from REC files and the characteristics of clinical projects (76% of all submissions) were analyzed.

**Results:**

The number of clinical projects was large, but declining: 794 in 2002 and 762 in 2007. Drug research (mainly trials) represented 29% and 34% of the clinical projects; their total number had not declined, but those without a commercial sponsor had. The number of different principal investigators was large (630 and 581). Most projects were observational, while an experimental design was used in 43% of projects. Multi-center studies were common. In half of the projects, the main funder was health care or was done as unpaid work; 31% had industry funding as the main source. There was a clear difference in the type of research by sponsorship. Industry-funded research was largely drug research, international multi-center studies, with randomized controlled or other experimental design. The findings for the two years were similar, but a university hospital as the main research site became less common between 2002 and 2007.

**Conclusions:**

Clinical research projects were common, but numbers are declining; research was largely funded by health care, with many physicians involved. Drug trials were a minority, even though most research promotion efforts and regulation concerns them.

## Background

Clinical research is a key element in strengthening the scientific basis of health services. Academic institutions and researchers in North America, and later, in Europe have expressed concerns about clinical research, while various initiatives to overcome those problems have been undertaken [1-10]. Regardless of these concerns, clinical research or clinical trials as a research institution have not been extensively studied. Previously, we have mapped the situation in one European country, Finland, using various sources [11]. Our results suggested that the governance and funding of clinical research in Finland was fragmented.

The aim of this paper is to describe the volume and orientation of clinical research in the same country by using submissions made to ethics committees in 2002 and 2007: What kind of clinical research was proposed to the committees, by whom, who sponsored it and how did sponsorship relate to the characteristics of research. The research law in Finland requires that all clinical research has to be considered by either locally based official research ethics committees (RECs) or a central committee. That requirement gave us a good opportunity to map the situation regarding clinical research in the country as a whole.

Finland is one of the Nordic welfare states, with a large public sector. A comparison to five European countries showed that research and development (R&D) expenses in general were high in Finland, but much of that was due to R&D undertaken by corporations [11]. Connections between public and private research units were more common in Finland than in the comparison countries (Denmark, Ireland, the Netherlands, Norway and Switzerland) [12]. The Finnish health care is largely public and decentralized, guided by local politicians. The state regulates health care through laws and steering, with state subsidies, and retrospective control. Health service funding involves two intertwined systems: first, a community based, tax-funded area-based system covering most in-patient care and much of out-patient care, and secondly, a national health insurance covering part of private care, occupational care, drugs, travel, and sickness absence costs.

## Methods

The research law requires that all medical research has to be submitted to an official REC. The RECs are area-based and the handling REC is determined by the work place of the principal investigator (PI). All multinational drug trials have to be submitted first to the central committee, but it delegates most of these to the official RECs. Practically all clinical research is included in the definition of medical research. Before 2011 the ethics review system for non-medical research in Finland was voluntary and unsystematic, and the official RECs had also handled other types of research, such as nursing or psychological research, usually at the request of researchers or the research site.

All projects submitted for the first time in 2002 and 2007 to one of the 20 official RECs and their subcommittees in continental Finland or to the central committee, were analyzed. The years 2002 and 2007 were chosen as we will also be studying publication bias and wanted to have at least five years to follow-up on appearance of publications (until 2012).

For each project, standard data for describing it and how it was handled were collected by researchers from REC documents. Filing of project submissions varied by REC and REC-secretaries helped to locate the needed documents. Usually meeting notes and summary data from the original submissions were available; the full application was not usually available. These were either in paper or electronic format. Most data were collected on site, but some smaller RECs sent copies of the documents. Data were extracted in written form or coded by the data collector. Data were entered into an electronic form (PASW Statistics 18). Data included the professional characteristics of PIs, research sites, field of research, sponsor, research methodology, REC decision, and handling fee.

It was possible for a single project to have several submissions (those not decided on at the first submission and those having later amendments). Data were collected from each submission and later combined per project by identifying the same project by the title, and if needed the PI and the ethics committee code. In the analysis each project was counted only once.

Collected data were coded either on site or later using all available information (Appendix 1). The main funder was as given in the application to the REC and was coded during data collection into the categories: industry, non-commercial (public) fund, or health care. Health care funding includes state subsidies to hospitals to do research, work done within normal working hours, and unpaid work.

We first calculated what proportion of all projects were clinical research projects, and then analyzed the clinical projects only. Clinical research projects (called clinical projects in brief) were defined based on two variables (“Type of research” and “Main content”) categorized at the time of data collection using all available information (see Appendix 1 for details).

### Permissions and ethics

The whole project (MERGO Ethical review and administrative governance of clinical research) received a positive statement from the National Institute for Health and Welfare’s ethics committee (June 17, 2010, amendment Jan 27, 2011). To study the submissions of research projects to the RECs in 2002 and 2007, special permission was obtained from the Health Ministry (STM/4257/2008) with later additions for new researchers; the confidentiality requirements of the Health Ministry were followed.

## Results

In 2002 and 2007 the official RECs received 2,060 submissions for research projects (Table 1). Three-quarters of the projects were defined as clinical projects. The subsequent information concerns only these.

**Table 1:** **Proportions (%) of different type of medical research projects in Finland in 2002 and 2007; projects submitted to the official Finnish ethics committees**^**1**^

	**2002**	**2007**	**Total**	***P *value**^**2**^
**(n)**	**(1014)**	**(1046)**	**(2060)**	
Drug research	22.9	24.9	24.0	0.287
Other medical research^3^	64.7	57.3	60.9	0.001
Non-medical research^3^	11.7	16.7	14.3	0.001
Missing	0.7	1.1	0.8	
Total	100	100	100	
Clinical research	78.3	72.8	75.5	0.01
(n)	(794)	(762)	(1556)	

^1^Includes projects handled by the Central committee.

^2^Comparison of the two years (*t*-test); in calculation *P* values, projects with missing information were ignored.

^3^As defined in the research law (see Methods).

In both years, the number of clinical projects per REC varied notably, from 1 to 291 in 2002 and from 1 to 321 in 2007. Most clinical projects were handled by the five university hospital RECs, and only the central committee non-university hospital REC handled more than 15 projects per year.

The number of different PIs was 630 in 2002 (794 projects) and 581 in 2007 (762 projects) in 2007. Thus one PI could have submitted multiple clinical projects in one year and that occurred more often in 2007 compared to 2002. The maximum number of projects per PI was 8 in 2002 and 9 in 2007.

Even though the total number of research projects did not decline (Table 1), the number of clinical projects showed a small decline between 2002 and 2007 (Table 2). The number of drug research projects (mainly trials) did not decline (232 in 2002 and 259 in 2007), but there was a decline in other medical research projects. Drug research represented 29% and 34% of the clinical projects in the respective years (Table 2). More than half of the drug research projects were international (Table 2). Over half (55%) of the clinical projects dealt with treatment and 21% were classified as diagnostic projects or studied disease characteristics.

**Table 2:** **Description of clinical research projects in Finland in 2002 and 2007, proportions (%) of projects**^**1**^

	**2002**	**2007**	**Total**	***P *value**^**6**^
**(n)**	**794**	**762**	**1556**	
**Type**				
Domestic drug research	11.1	13.6	12.4	0.601
International drug research	18.1	20.4	19.2	0.615
Other medical research	70.8	66.0	68.4	0.090
**Main content**				
Treatment	54.0	56.6	55.3	0.443
Diagnostics and disease	24.0	18.0	21.1	0.192
Biomedical	8.7	5.6	7.2	0.545
Prevention	7.2	5.0	6.1	0.665
Other + missing	6.1	14.8	10.3	--
**Approach**				
Randomized controlled trial	15.9	23.8	19.8	0.092
Trial + intervention	24.3	21.1	22.7	0.475
Observational	43.7	41.5	42.6	0.567
Documents + registers	4.8	6.2	5.5	0.780
Other + missing	11.3	7.4	9.4	--
**Main funder**^**2**^				
Industry	28.7	33.7	31.2	0.236
Public fund	16.3	16.0	16.1	0.966
Health care	52.1	42.3	47.3	0.010
Missing	2.9	8.0	5.4	--
**Data collection site**^**3**^				
University hospital	63.8	43.7	54.0	<0.001
Other public health care facility	13.5	20.1	16.7	0.165
Private care	7.5	7.4	7.4	0.984
Other place	10.9	14.7	12.8	0.417
Missing	4.3	14.1	9.1	--
Total	100	100	100	
**Non-commercial**^**4**^	75.4	70.9	73.2	0.087
**Multi-center study**^**5**^				
National	27.8	30.7	29.2	0.497
International	21.3	23.8	22.5	0.576

^1^For the explanations of the categories, see Appendix 1.

^2^As given in the application. Health care includes state subsidy to hospitals to do research, work done within normal working hours or on own time.

^3^In multi-center studies, the place of the principal investigator was chosen for the main research site.

^4^Administratively non-commercial i.e. exempted from REC fee.

^5^A project can be both national and international multicenter project.

^6^Comparison of the two years (*t*-test); in calculation *P* values, projects with missing information were ignored.

The proportions of different types of clinical projects in the two years were relatively similar, but there were more clinical projects in 2007 that could not be classified on the basis of the information in the documents. If those were excluded from the denominator, the proportions of treatment projects somewhat increased (from 58% to 66%, *P* ≤0.01) and diagnostic/disease somewhat declined (26% and 21%, *P* = 0.07).

The most common approach used was observational, in which patients or research subjects were studied without exposing them to research interventions (Table 2). We classified 43% as experimental (trials) having either a comparable or non-comparable control group or without an explicit control group. The share of randomized controlled trials (RCTs) somewhat increased between 2002 and 2007 (from 16% to 24%) even though this was not statistically significant (Table 2).

The most common data collection site (in multi-center studies the place where the Finnish PI collected the data) was a university hospital, but this was less so in 2007 (Table 2). Other public health care facilities, particularly central hospitals other than university hospitals, had more projects in 2007. However, in 2007 the information on the data collection site was lacking for more projects than in 2002, making the comparison potentially unreliable.

Usually the main data collection site was also the work place of the PI (as given in the application), but not always. Finnish physicians can work in both public and private care at the same time. We cross-tabulated the data on the collection site and the work place of the PI. Out of 849 projects in which the work-place of PI was defined as a university central hospital, in 14% the main data collection site was elsewhere, particularly in other central hospitals (7.3%); 1.2% were in private care. Out of 238 projects in which the work-place of the PI was defined as university or other public research institution, in 58% of the projects the main data collection site was elsewhere, most commonly (25%) in a university hospital.

In both years domestic and international multi-center studies were common (Table 2). Overall, 35% in 2002 and 39% in 2007 were multicenter studies. We did not collect systematic information on what kinds of institutions participated in multi-center studies as partners.

The most common main funder was “health care” (Table 2), which meant that research was mainly paid for by health care, including through state subsidies to hospitals, work done within normal working hours, or unfunded research (done on own time). In about a third of the projects the main funder was industry. However, the documents specified clearly only the main funder, and very likely many projects were funded from several sources. The proportions in the two years were relatively similar, but there were more projects in 2007 that could not be classified on the basis of the information in the documents used (Table 2).

Administratively, non-commercial projects were defined on the basis of whether the research ethics committee (REC) had exempted the study from the handling fee. In 2002, 75% and in 2007, 71% of the projects were exempted (Table 2). Practically all projects funded by a public fund or health care were exempted, but also 21% of industry funded projects in 2002 and 16% in 2007 were exempted (Table 3). It is likely that these were projects that received some funding from industry (*e.g.*, donations of drugs or medical devices), but which were not fully covered by industry.

**Table 3:** **Relation of the characteristics of clinical research projects to funding, projects submitted to the official Finnish ethics committees in 2002 and 2007, %**^**1**^

**(n)**	**2002**				**2007**			
	**Industry**	**Public**	**Health care**^**4**^	***P *value**^**6**^	**Industry**	**Public**	**Health care**^**4**^	***P *value**^**6**^
	**(228)**	**(129)**	**(414)**		**(257)**	**(122)**	**(322)**	
**Type**								
Domestic drug research	20.2	6.2	7.5	<0.001	25.7	6.6	8.1	<0.001
International drug research	58.8	1.6	1.4	<0.001	57.6	4.1	0.6	<0.001
Other medical research	21.1	92.2	91.1	<0.001	16.7	89.3	91.3	<0.001
**Main content**								
Treatment	80.3	23.2	48.3	<0.001	87.5	33.6	46.0	<0.001
Diagnostics and disease^2^	4.8	31.8	33.6	<0.001	2.7	23.8	28.8	<0.001
Biomedical^3^	7.0	23.3	5.6	0.478	3.1	16.4	4.7	0.328
Prevention	6.1	15.5	5.3	0.673	2.7	13.1	4.7	0.212
Other + missing	1.8	6.2	7.2		3.9	13.1	15.8	
**Approach**								
RCT	43.0	7.8	3.9	<0.001	59.9	6.6	4.3	<0.001
Trial + intervention	36.0	14.7	20.0	<0.001	28.8	20.5	16.2	<0.001
Observational	16.2	59.6	54.1	<0.001	5.8	56.6	61.5	<0.001
Documents + registers	1.3	3.1	7.6	<0.001	2.7	5.7	8.4	0.004
Other + missing	3.5	14.8	14.4		2.8	10.6	9.6	
**Data collection site**^**2**^								
University hospital	57.0	56.6	72.5	<0.001	38.5	38.5	53.4	<0.001
Other public health care facility	9.6	16.4	15.5	0.486	16.7	18.9	26.7	0.760
Private care	16.3	3.8	2.4	<0.001	17.9	3.3	1.7	<0.001
Other place	14.0	21.6	6.2	<0.001	19.9	22.1	9.2	<0.001
Missing	3.1	1.6	3.4		7.0	17.2	9.0	
Total	100	100	100		100	100	100	
**Non-commercial**^**3**^	20.6	96.1	98.8	<0.001	15.6	98.4	99.1	<0.001
**Multi-center study**^**5**^								
National	46.1	34.9	16.4	<0.001	46.7	33.6	22.0	<0.001
International	61.0	9.3	3.6	<0.001	59.1	12.3	3.1	<0.001

^1^For the explanations of the categories, see Appendix 1.

^2^In multi-center studies, the place of the principal investigator was chosen for the main research site.

^3^Administratively non-commercial i.e. exempted from REC fee.

^4^Health care includes state subsidy to hospitals to do research, work done within normal working hours or on own time.

^5^A project can be both national and international multicenter project.

^6^Comparison of industry funded projects to health care funded projects (*t*-test); in calculation *P* values, projects with missing information were ignored.

Table 3 describes the characteristics of the clinical projects by the main funder. There was a clear difference in the type of research by sponsorship. Industry funded research was largely drug research and concerned treatment, with most being RCTs or other types of experimental designs. Publicly funded and health-care-funded research was rarely drug research and very few were RCTs, but some had other experimental designs; the most common approach was patient follow-up (observational studies). The main content was more varied than in industry funded research, particularly in projects funded by public grants. Health-care-funded projects were most commonly treatment and diagnostic/disease projects.

Of the 232 drug research projects in 2002 and 259 projects in 2007, 79% and 83%, respectively, were funded by industry. Most industry funded research was multi-center studies, usually international (*i.e.*, including centers outside Finland). Also, projects with public funding were frequently multi-center studies, though more often domestic. Health-care-funded research was less frequently multi-center, and when it was, it was mainly domestic to Finland.

Results for the two years were similar with regards to type, main content, multicenter and non-commercial status. With regards to approach and the data collection site, results differed across the two study years. Drug trials sponsored by industry somewhat increased between 2002 (n = 180) and 2007 (n = 214), but those without a commercial sponsor did not (n = 47 and 41). In industry sponsored projects, RCTs became more common between 2002 and 2007, but not in other projects (Table 3).

In 2002 the main data collection site was a university hospital in all types of research, but that had changed by 2007. In 2007, 39% of industry sponsored projects were conducted in university hospitals; projects in other public health care facilities had become more common (Table 3). The share of projects conducted in private health care settings (16% and 18%) remained the same. University hospitals had also become less common study sites for public grant and health-care-funded projects, even though among health-care-funded projects they were still the most common site (53%). If projects that lacked information on the study site are ignored, 75% of health-care-funded projects were conducted in university hospitals in 2002 and 59% in 2007 *(P <*0.001).

## Discussion

The number of clinical research projects submitted to the Finnish research ethics committees in the two sampled years was high, but declined between 2002 and 2007, even though the total number of medical research projects did not. Most clinical research projects were studies on treatments, diagnosis, or disease mechanisms, and about half were observational. Most projects were non-commercial. Thirty-two percent of the clinical projects concerned drugs and their numbers did not decline during the study period. The number of drug projects with commercial sponsorship increased somewhat, but those without a commercial sponsor did not. The number of different PIs was high. Most projects were health care funded or done as unpaid work. University hospitals were the most common research sites, but declined between 2002 and 2007, particularly for industry funded research. Those funded by industry differed from the rest of the projects in terms of all the studied characteristics, while also being concentrated on drug trials. Industry sponsored projects were often RCTs. The results by sponsor in the two studied years were mostly similar, but some differences by the funder became larger over time.

### Comments on findings

We found little comparable data from other countries. In the Netherlands, national statistics from accredited ethics committees on medical research have been published in English for 2010 [13]. In addition, we comment on the findings and compare our results, where feasible, to two studies from the US [1,14]. However, the data from the US studies came from a trial register (clinicaltrials.com), and they do not refer to a defined period of time (accumulation of studies started in 2000s) or place (several countries are partly covered) and are thus not strictly comparable.

The number of clinical research projects in the sampled years was high, but declining. In our previous study [11] we reported data from national statistics, concluding that the quality and quantity of Finnish clinical research had been high compared to some other countries, but volume had declined in the 2000s [15]. One possible factor that reduced the number of clinical projects is the European Clinical Trials Directive, which came into force in 2004 in Finland [16]. Another reason may be the tighter budgeting and oversight of clinical research in health care units, which has made the real costs visible as well as reducing the number of poorly planned projects. However, we did not have data on the quality of the projects and do not know whether the share of poorly planned studies has declined.

The characteristics of clinical projects in the two studied years were relatively similar, as one might expect with a 5-year time difference. However, some findings suggest a changing scene in clinical research: the total number of clinical projects sponsored by industry had not declined, but those funded by health care had. Accordingly, commercial drug research did not decline. Industry funded projects differed from the rest in all aspects that were examined. RCTs became more common between 2002 and 2007 in industry sponsored projects, but not in others. University hospitals served as the main research site less often. These changes may reflect declining state subsidies for clinical research in health care [16], in addition to increased work pressure for clinicians in public hospitals.

Finnish regulations require that in the case of medical studies, each study site has to have a responsible researcher with a medical education. During the study years the number of different PIs was large and a large number of the projects were multi-center studies that also involved other researchers. However, the number of different PIs declined between 2002 and 2007, and the number of projects per single PI increased. In a 2006 survey, a high proportion of physicians reported engagement in research (clinical research was not specified), but later surveys suggested that in many hospitals and health centers, the possibilities to undertake clinical research were deteriorating, particularly due to the burden of routine patient care and a lack of time [11].

Data were not available on whether researchers were continuously engaged in research over time. A study on clinical trials from the US [1] showed a high turnover of clinical investigators. In 2007, 26,000 investigators were registered with the FDA for drug trials, with most of them registered only for a single trial; foreign investigators have been replacing domestic ones [1].

According to our summary data, the PI was most commonly from a university hospital. However, this may not reflect the actual circumstances. The recording system of RECs very likely inflates the importance of university hospitals due to the tradition of local decision-makers allocating government subsidy money to favor established researchers from university hospitals as PIs instead of other co-researchers. Second, projects in the medical schools and their biotechnology units and the projects may have been anchored to the hospital rather than the university in order to be eligible for EVO-funding. Third, many university hospital physicians have sideline work in the private sector and the actual study may have been done there. The web of public, semipublic and private units has expanded rapidly, and the information demands in clinical research regulation have not followed this development.

In about half of the projects the main funder was “health care”, which indicates a mixture of funding. Municipalities allowed health professionals to use their working hours for clinical research, but they did not directly fund clinical research [11]. The work time compensation could be an important support for encouraging research, but in practice it is rather limited as the health professionals’ work-load often prevents research during working hours. The national health insurance (KELA) has research funds, but their focus has not been on clinical research [11].

We did not have data on project size and costs, so small projects weighed as much as large projects in our data. Cost data are very difficult to find, as budgeting for and reporting on research expenses is different for different sponsors, and varies by the type of institute. The documents only identified clearly the main funder, and it is possible that external money was more frequently designated as the main funding source. This makes the contribution of the public sector appear smaller than it is, as much of their contribution has been in work time and the creation of infrastructure. On the other hand, anecdotal evidence suggests that in commercial projects the amount of visible money (separately in accounts books) is relatively larger than the number of projects. It may be that government and municipality funding has been used to create the infrastructure for clinical research, but external funding determines more what types of projects are undertaken; this increases the role of industry in directing research.

In the Netherlands [13], RECs handled 702 submissions for medical research in 2010. However, we could not extract the clinical research or change the submissions into projects to correspond our study, which is a drawback in the subsequent comparisons. Unlike Finland, most (58%) of the submissions concerned trials, *i.e.*, research with an experimental design. The proportion of multicenter studies (38%) was similar to that in Finland. Like in Finland, about a third concerned drugs, but the share (47%) sponsored by non-industry sources was higher than in Finland, where the proportion of drug projects funded by non-industry sponsors was 16% in 2007. In the Netherlands, 42% of drug projects were either phase I or phase II drug trials.

In the American study using trial registration data for trials initiated in the 2000s, it was found that most trials concerned drugs (59%) and half of them concerned cancer [1]. A later study using the same registration database found that, similar to our study, most trials in terms of numbers were sponsored by academic health centers and were small [13]. However, when the number of patients was studied, most were from industry sponsored phase 3 and 4 studies. Likewise in the earlier US study [1], two-thirds (63%) concerned drugs.

### Drug trials

We found that about a third of the clinical projects concerned drugs and overall their numbers had not declined. However, those drug trials without a commercial sponsor declined somewhat. This is in accordance with the statistics of the drug authority: the total number of started clinical drug trials the years 2001 and 2006 fluctuated between 238 and 293 declining to 209 trials in 2010, with the largest decline in phase 2 and phase 1 trials [17]. There was a decline in the number of trials without an outside commercial sponsor (academic projects): about 90 in 2002 and 40 in 2010 [17].

A study comparing six European countries found a notable variation in the trends of the total number of drug trials and academic drug trials from 2001 to 2009 [18]. For example, the total number of drug trials increased in Italy and in Spain; in Italy due to academic trials and in Spain due to commercial trials. In UK, there was a sharp decline in the number of academic trials.

Clinical projects funded by industry have concentrated on drug trials. This is not unexpected, but raises the question of data balance [19-22]. Light et al. [23] claim that clinical trials in North-America have become a high-profit sub-industry, largely carried out by for-profit contract research organizations (on behalf of the industry).

In our study years the majority of clinical research projects were not related to drug research, even though regulation is centered on drug research [11]. Similarly, outside Finland there has been a special focus on drug trials due to the drug industry’s commercial interests and lobbying power, and in a European context, also due to the EU mandate. It has been said that the European Clinical Trials Directive (in force since 2004) makes drug trials more difficult to undertake and has specifically decreased the number of trials sponsored by non-commercial actors [16,24-26]. Hope for reducing these problems has now been put in modification of the Clinical Trials Directive, which is currently (Spring 2013) under discussion (Piret et al., unpublished manuscript).

### Methodological comments

The data from each project at our disposal were limited and for some variables more information was lacking in 2007. Our classification of clinical research as a sub-class of medical research was subjective. Various definitions and terms have been used for clinical research and there has been no uniform definition that can be applied. One could argue that patient-oriented research by paramedical researchers such as psychologists, which we excluded, could be defined as clinical research. However, most projects were easy and clear to classify as clinical research, and we can confidently consider the data to be representative of clinical research with proposed start dates in 2002 and 2007 in Finland.

We also undertook a number of other classifications (see Appendix 1). Mostly coding was easy and clear cut, but the complexity of each project was difficult to compress into the simplified codes we used. For example, many projects received funding or other resources from different sources, but only one, apparently the main one, was indicted in the summary data. More detailed coding was not considered useful, as for most projects the data we had at hand were limited.

A strength of our study is that it covers a defined time period and the entire country. This was due to the medical research law requiring all medical research (including clinical research) to be handled in official research ethics committees. We needed special permission to access the data that are currently considered confidential. The permissions and practices were cumbersome and took a significant amount of time. The data available in research ethics committees were useful to us and could also be for others interested in research policy and outcomes. To facilitate such research, a change in attitudes and interpretation of what is public and what is confidential would be welcome.

## Appendix 1

Classifications and coding made.

**Type (Medical research):** In the data collection phase each project was classified into four groups (domestic drug research, international drug research, medical research, other research) by the help of the information in the (summary) study plan and the classification used by the REC. Medical research is defined by the law: “Medical research means research involving intervention in the integrity of a person, human embryo or human foetus for the purpose of increasing knowledge of health, the causes, symptoms, diagnosis, treatment and prevention of diseases or the nature of diseases in general.” However, RECs might have adapted that definition in their classification.

**Content (Type of research):** The type of research had 14 categories, which were defined at the time of data collection using all available information. The categories were combined for this paper into: Treatment (6 patient treatments), diagnostics and disease (3 disease classes, 8 diagnostics), biomedical (9 biomedical research), prevention (1 prevention, 2 epidemiology), other (4 dentistry, 5 health behavior, 7 health services, 10 psychology, behavioral and social sciences, nutrition, etc., 11 technology, 12 other, 13 nursing studies, 14 sport medicine). Biomedical research covers projects in pharmacology, microbiology, biochemistry, etc. using human samples to understand physiological and pathological mechanisms and not focusing on patient outcomes.

**Clinical research:** Clinical projects were defined by cross-tabulating variables “Type” and “Content”. Under “Type” classes, “drug research” and “other medical research” were picked, excluding “other research” and missing. “Other medical research” was then cross-tabulated by “Content”; based on that cross-tabulation projects that were defined as epidemiology, health behavior or psychology were excluded.

The categories of clinical research were combined for this paper into: Treatment (6 patient treatments), diagnostics and disease (3 disease class, 8 diagnostics), biomedical (9 biomedical research), prevention (1 prevention, 2 epidemiology), other (including 4 dentistry, 5 sport medicine, health services research).

**Approach:** Each project was coded afterwards into nine categories using the project title, method of research, type of research and EudraCT code (yes or no), and other information in the original data collection file. The categories were combined for this paper into: RCT, other trial and intervention (includes 2 other trial, 3 intervention, *i.e.*, having an experimental design), observational (4 clinical follow-up study, 5 non-clinical follow-up, 6 not clear), documents and registers (7 a study based on ready documents only; includes follow-up studies of trials using documents only), and other (8 other; includes quality assurance).

**Principal investigator:** The responsible researcher in Finland, as in REC application.

**Work place:** of the principal investigator, as in REC application. The coding was made as for “Data collection site”.

**Data collection site:** The site was coded from the written name of the places where the research was to be made. If the name was not known to the coder, Google search was used to get further information. The following classes were used: 1 university hospital, 2 other central hospital, 3 district hospital, 4 other public hospital, 5 private hospital, 6 private outpatient clinic, 7 public health center, 8 occupational health, 9 non-profit, private, 10 university or research center, 11 polytechnic, 12 industry, private research center, 13 other. Classes 2–4 and 7 were combined to other public health care facility; 5 + 6 + 8 + 9 were combined to (other) private care, 10–13 to other place; there were very few projects in option 8.

In multi-center studies, the place of the principal investigator was chosen for the main data collection site. In case of lacking information, the work place of the principle investigator was used, if it was available (5% of projects).

**Domestic multi-center study:** At least two study sites in Finland.

**International multi-center study:** At least one study site was abroad.

**Main funder:** Main funder as given in the application to REC, coded during data collection into: industry, non-commercial (public) fund, health care. Health care includes state subsidy to hospitals to do research, work done within normal working hours, and as unpaid work.

**Non-commercial project:** the projected is exempted from REC fee.

## Abbreviations

PI: Principal investigator; RCT: Randomized clinical trial; REC: research ethics committee.

## Competing interests

The authors declare that they have no competing interests

## Authors’ contributions

EH originated the idea, planned the analysis and prepared the draft manuscript. PV participated in designing the study and commented on the manuscript. JV participated in designing the study, collected part of the data and commented on the manuscript. ER carried out the analysis and commented on the manuscript. All authors read and approved the final manuscript.
